# Cetuximab promotes epithelial to mesenchymal transition and cancer associated fibroblasts in patients with head and neck cancer

**DOI:** 10.18632/oncotarget.5924

**Published:** 2015-09-30

**Authors:** Sandra Schmitz, Gabriela Bindea, Roxana Irina Albu, Bernhard Mlecnik, Jean-Pascal Machiels

**Affiliations:** ^1^ Institut Roi Albert II, Department of Medical Oncology, Cliniques Universitaires Saint-Luc and Institut de Recherche Clinique et Expérimentale (Pole MIRO), Université catholique de Louvain, Brussels, Belgium; ^2^ Department of Head and Neck Surgery, Cliniques Universitaires Saint-Luc, Université catholique de Louvain, Brussels, Belgium; ^3^ INSERM UMRS1138, Laboratory of Integrative Cancer Immunology, Paris, France; ^4^ Université Paris Descartes, Rue de l'Ecole de Médecine, Paris, France; ^5^ Cordeliers Research Centre, Université Pierre et Marie Curie Paris, Paris, France

**Keywords:** head and neck cancer, epithelial-mesenchymal transition, cancer-associated fibroblasts, anti-EGFR therapy, pharmacodynamic

## Abstract

**Purpose:**

To investigate if cetuximab induces epithelial to mesenchymal transition (EMT) and activation of cancer associated fibroblast (CAF) in the tumors of patients with squamous cell carcinoma of the head and neck (SCCHN).

**Methods:**

Cetuximab was administered for two weeks prior to surgery to 20 treatment-naïve patients. Five untreated patients were included as controls. Tumor biopsies were performed at baseline and before surgery. Gene expression profiles and quantitative real-time PCR (qRT-PCR) analysis of the pre-and post-treatment biopsies were compared. To further investigate EMT and CAF, correlations between previously described EMT and CAF markers and our microarray data set were calculated.

**Results:**

Gene expression profile analyses and qRT-PCR showed that some of the genes modified by cetuximab were related to CAFs and EMT (*ZNF521*, *CXCL12, ASPN, OLFML3, OLFM1, TWIST1, LEF1, ZEB1, FAP)*. We identified 2 patient clusters with different EMT and CAF characteristics. Whereas one cluster showed clear upregulation of expression of genes implicated in CAF and EMT including markers of embryologic pathways like NOTCH and Wnt, the other did not.

**Conclusion:**

Even if EMT and CAFs are implicated in cetuximab resistance in pre-clinical models, we demonstrate for the first time that these molecular processes may occur clinically early on.

## INTRODUCTION

With an incidence of over 650.000 people per year, squamous cell carcinoma of the head and neck (SCCHN) is the sixth most common cancer worldwide. Despite combined treatment in patients with stage III/IV SCCHN, long-term survival rates remain low in advanced stages, and chemoradiation regimens have reached their upper limit of tolerability [1]. Therefore, the investigation of new targets, based on the biology of the disease, is of major importance.

Cetuximab is recommended as treatment for patients with locally advanced SCCHN. This chimeric IgG1 monoclonal antibody (mAb) specifically binds to the epidermal growth factor receptor (EGFR) with high affinity. EGFR is a transmembrane tyrosine kinase receptor belonging to the HER/erbB family. Up to 90% of SCCHN express high levels of EGFR [2]. The overexpression of EGFR and tumor growth factor-alpha (TGF-α) is associated with poor prognosis [3, 4] and radioresistance [3, 5].

Cetuximab improves the overall survival when associated with radiation therapy in locally advanced SCCHN, or with platinum-based chemotherapy in incurable disease [6, 7]. However, with single agent objective response rates between 6% and 13%, only a minority of patients benefit from anti-EGFR mAbs [7, 8]. In contrast to colon cancer, where *RAS* mutations predict treatment resistance [9], little is known about the potential mechanisms of cetuximab resistance in SCCHN. Hypotheses to explain anti-EGFR resistance include the acquisition of oncogene activating mutations, activation of alternative signaling growth pathways, or modifications in tumor composition [10-12].

Components of the tumor microenvironment have been implicated in treatment resistance and have been shown to influence tumor growth and progression [13-15]. Epithelial to mesenchymal transition (EMT), a process in which cancer cells lose cell-to-cell adhesion and gain invasive properties, has been described in preclinical models, including SCCHN, as a possible mechanism of EGFR therapy resistance [11]. Cancer-associated fibroblasts (CAFs), essential components of the tumor microenvironment, may also modulate EGFR treatment sensitivity and promote tumor growth [12]. Evidence of this in real clinical situations is, however, lacking.

We have previously shown that pre-operative administration of cetuximab in a window opportunity study was safe and active [16]. We extended the study to compare pre- and post-cetuximab tumor biopsies for gene and protein expression. We show here that cetuximab has a strong anti-tumoral effect, but that it also induces significant modifications in the tumor that may promote subsequent tumor growth and aggressiveness.

## RESULTS

### Clinical results

Cetuximab was administered for two weeks prior to surgery to 20 treatment-naïve SCCHN patients selected for surgical treatment, as previously reported [16]. Ninety percent of the patients achieved a 18FDG-PET response, but the majority had no significant macroscopic tumor shrinkage. In patients treated with cetuximab, histopathological analysis of the tumor specimens at surgery showed reduced tumor cellularity, downregulation of tumor proliferation and decreased phospho-EGFR compared to untreated controls [16].

### Cetuximab monotherapy induced modifications in tumor composition

**Figure 1:**
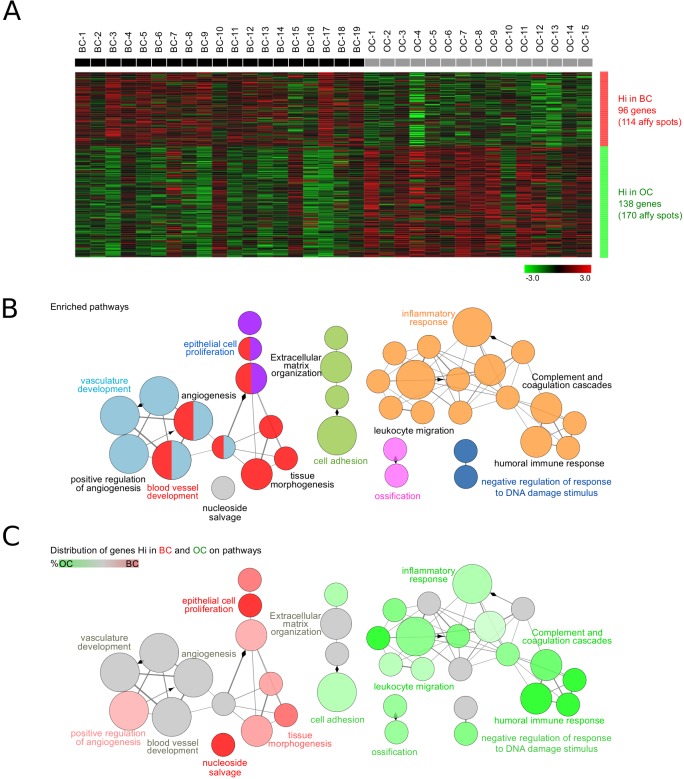
Biological role of the differently expressed genes between the pre- and post-treatment tumor biopsies **A.** Gene expression in biopsies taken at baseline (BC, *n* = 19) and under operative (OC, *n* = 15) conditions was investigated. Upregulated genes having at least two times higher mean expression (delta >1) per group compared to the others were selected. The expression of significantly differentially expressed genes (*P* < 0.05) was normalized and visualized in Genesis. BC and OC are coloured in black and grey, respectively. Genes with high (Hi) and low (Lo) expression are shown in red and green, respectively. **B.** ClueGO functional analysis of the 104 genes (284 Affymetrix spots) significantly differentially expressed between OC and BC. Gene Ontology, KEGG and Reactome pathways were used. Functionally grouped network with pathways and terms as nodes linked based on their kappa score. The node size represents the term significance. Fusion was applied to remove redundant terms. Only pathways with *P* < 0.05 after Benjamini correction for multiple testing were included in the network. Functionally related groups partially overlap. Not grouped terms are shown in grey. **C.** Distribution of the differently expressed genes in BC (*n* = 96) and OC (*n* = 138) on the network of pathways from **B.**. Terms with high expression genes in BC and OC are shown in red and green, respectively. The color gradient shows the gene proportion of each cluster associated with the term. Equal proportions of the two clusters are represented in grey. Pathways and associated genes are presented in Supplementary Figure. 1.

Initially, the impact of cetuximab on gene expression was obtained through a global analysis of the Affymetrix gene expression data with the aim of determining which genes are differently expressed between the pre- and post-treatment biopsies, and in which biological processes they are involved. The expression data from the baseline biopsies (BC) and the operative biopsies (OC) was compared. Only high quality samples were used for the analysis (*n* = 19 before and *n* = 15 after cetuximab). Upregulated genes, with at least two times higher mean expression (delta >1) per group compared to the others, were selected. Out of these, 284 Affymetrix spots were significantly differentially expressed (*P* < 0.05) between the BC and OC (Supplementary Table 1). The expression of 96 genes (114 spots) upregulated in the BC compared to the OC, and of 138 genes (170 spots) significantly more highly expressed in the OC, was normalized and visualized in Genesis (Figure 1A).

ClueGO [17] functional analysis revealed that these genes were involved in pathways associated with angiogenesis, epithelial cell proliferation, cell adhesion, extracellular matrix organization, inflammatory response and negative regulation of response to DNA damage stimulus (Figure 1B). Interestingly, the 96 genes downregulated by cetuximab were mainly associated with epithelial cell proliferation, tissue morphogenesis, positive regulation of angiogenesis, and nucleoside salvage. In contrast, the 138 genes upregulated after cetuximab were specifically involved in extracellular matrix organization, cell adhesion and immune-related domains such as inflammatory response, humoral immune response, complement and coagulation cascade, and leukocyte migration (Figure 1C, Supplementary Figure 1A).

Among the genes differently expressed, 41 had a strong fold change in their expression (an absolute fold change of 1.8 i.e. 0.85 Log2 scale, and a corrected *P* for multiple testing with a Benjamini-Hochberg test (BH) below 5%).

Six of these genes were associated with inflammation: *ADAMDEC1, APOC1, C3, CD163, ACKR1, VSIG4*. Another 20 genes are known to be implicated in cancer-related processes.

Some of these cancer-related genes were associated with processes occurring in the extracellular matrix and related to *DCN*: *CXCR4/CXCL12 axis, SELP, vWF, COL3A1, SCARA, SPARCL1*. Other genes were related to CAFs and EMT (ZNF521, *CXCL12, ASPN, OLFML3, OLFM1*) (Supplementary Table 2).

Following cetuximab treatment, the overexpression of selected genes related to either cancer stroma processes or CAFs, and EMT, was confirmed by quantitative real-time PCR (qRT-PCR) and immunochemistry (IHC). A significantly higher expression of *DCN* (*P* = 0.0010)*, SPARCL1* (*P* = 0.0207), *OLFM1* (*P* = 0.0010), *OLFML3* (*P* = 0.0016), and *CXCL12* (*P* = 0.0011) was observed after cetuximab but not in the biopsies of the untreated patients (data not shown). A good correlation was obtained between the Affymetrix and qPCR measured gene expression if the genes had a higher expression than a Log2 intensity of 3 in the Affymetrix data (e.g. r = 0.84 for *OLFM1*), confirming the reproducibility of the microarray-based results. IHC staining on the resected tumor specimens confirmed the upregulation of decorin in the cetuximab treated group when compared to the control group (*P =* 0.0068) (Supplementary Figure 1B and 1C). In addition, we found inverse correlations between decorin expression, measured by qRT-PCR or IHC, and the residual tumor cellularity of the surgical specimens (r = −0.64, *P* = 0.0006; and r = −0.55, *P* = 0.0067, respectively), as well as with 18FDG/PET response, the primary endpoint of our clinical trial (r = −0.50, *P* = 0.0120; and r = −0.4380, *P* = 0.0207, respectively) (Supplementary Figure 1D, 1E and 1F). P21WAF1/CIP1, a potent inhibitor of cyclin-dependant kinases, has been shown to be upregulated after the interaction between decorin and EGFR. P21 overexpression was also demonstrated after cetuximab by IHC (Supplementary Figure 1G) but was not found in the untreated samples (data not shown).

### Cetuximab induced EMT

**Figure 2:**
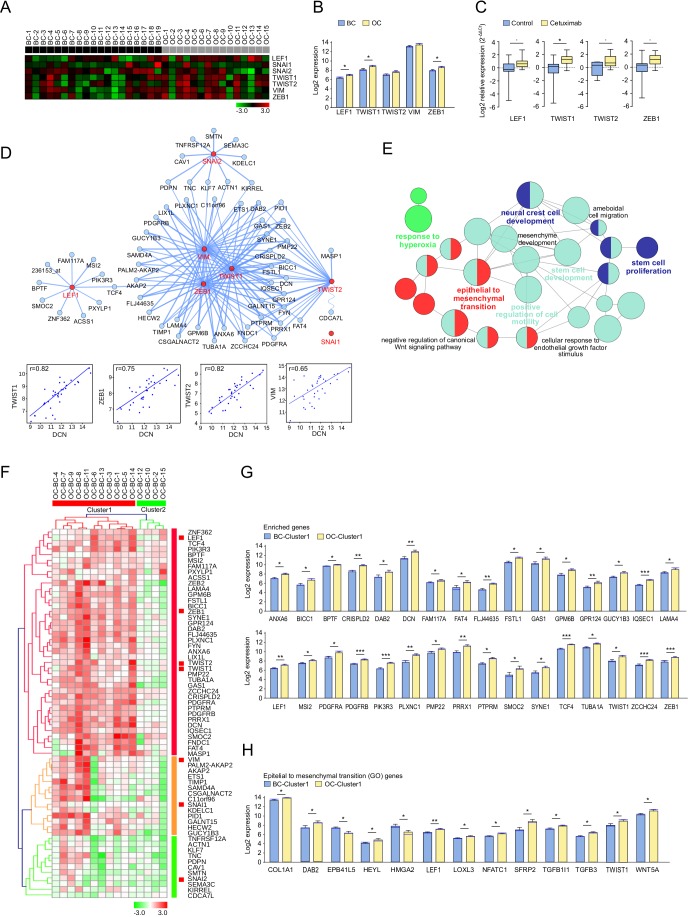
Expression pattern of epithelial to mesenchymal transition (EMT) markers and related genes after treatment with cetuximab in SCCHN patients **A.** The expression of known EMT related genes in biopsies taken at baseline (BC, *n* = 19) and under operative (OC, *n* = 15) conditions. BC and OC are coloured in black and grey, respectively. Data was normalized. Genes with high and low expression are shown in red and green, respectively. **B.** Affymetrix measured EMT marker expression in OC (yellow) and BC (blue). Bar charts represent the mean (± SEM), and the median expression is shown in blue. Wilcoxon-Mann-Whitney test was applied (* 0.01≤ *P* < 0.05). **C.** Relative RNA expression levels (RT-qPCR) of EMT markers in OC (yellow) and BC (blue). Whiskers represent means ± SD. **D.** CluePedia network showing EMT markers (red) together with the top 10 correlating genes with each of these markers (blue). SNAI1 had low expression (< 3). The edges represent Pearson r values (*P* > 0.6). Negative correlations are shown with a sinusoidal line. Correlation examples are shown for DCN and EMT makers. **E.** ClueGO functional analysis of the EMT markers and enriched genes described at **D.**. Functionally grouped network with terms as nodes linked based on their kappa score. The node size represents the term enrichment significance. Fusion was applied to remove redundant terms. Only pathways with *P* < 0.05 after Benjamini correction for multiple testing were included in the network. Functionally related groups partially overlap. **F.** The fold change expression (OC-BC) of EMT markers and enriched genes described at **D.** in SCCHN patients with complete time points (*n* = 15). The fold change matrix was normalized and hierarchical clustered (Kendall's tau, Average Linkage). Two patient clusters were revealed. Increased and decreased expression in OC compared to BC is shown in red and green, respectively. Significantly increased expression after cetuximab treatment of **G.** EMT markers and enriched genes described at **D.**, as well as of **H.** genes associated to the epithelial and mesenchymal transition pathway from Gene Ontology (GO), in patients from cluster 1 **F.**. Bar charts represent the mean (± SEM) in OC (yellow) and BC (blue), and the median expression is shown in blue. Wilcoxon-Mann-Whitney test was applied (* 0.01≤ *P* < 0.05, ** 0.001≤ *P* < 0.01, ***P < 0.001). Genes with a similar trend can be seen in Supplementary Figure 2.

In the second phase of this study, a detailed analysis aimed at investigating the impact of cetuximab treatment on the EMT process was performed. Seven genes, known to be related to EMT in SCCHN (*ZEB1, TWIST1, TWIST2, LEF1, VIM, SNAI1 and SNAI2*) [18, 19], were analyzed. Their expression was normalized and visualized in Genesis (Figure 2A). Interestingly, the known EMT markers *LEF1*, *TWIST1*, and *ZEB1*, showed significantly increased expression in post-treatment biopsies (Figure 2B). Increased expression in OC was observed for *TWIST2* and *VIM*. *SNAI1* was minimally expressed in the microarray (expression <3), and therefore filtered out for further analysis. These results were confirmed by qPCR in the BC and OC samples of cetuximab treated and untreated patients. A significantly higher expression was observed for *TWIST1* (*P* < 0.05) (Figure 2C). To investigate which other genes behave in a similar way after cetuximab treatment, correlations between the six selected EMT genes and all the other Affymetrix tested genes were calculated with CluePedia [20]. The top 10 genes, with the highest correlation (r > 0.6) with each of the six markers, were found to be enriching the EMT known marker network (Figure 2D). Interestingly, *DCN* was also found among the EMT enriched genes, which positively correlated with *TWIST1* (r = 0.83), *TWIST2* (r = 0.83), *ZEB1* (r = 0.76) and *VIM* (r = 0.65). ClueGO functional analysis revealed that these genes are involved in pathways relating to EMT, stem cell proliferation, and the development and positive regulation of cell motility (Figure 2E).

The expression of the EMT enriched genes was heterogeneous in patients with a complete set of time points (data not shown). The unsupervised clustering of the fold increase or decrease in OC compared to BC revealed two patient groups with different EMT pathway related modifications. Overall, cluster 1 patients had a significantly increased expression of EMT-related genes (*P* < 0.0001), compared to cluster 2 patients. The fold change visualization thus allowed the identification of patient groups with similar changes in EMT-related gene expression after cetuximab treatment (Figure 2F, Supplementary Table 3). Statistically significant (*P* < 0.05) gene modifications between both time points are shown in Supplementary Figure 2a for all patient samples, and in Figure 2G for patients cluster 1. Genes with a *P* - value in between 0.05 and 0.1 are represented in Supplementary Figure 2B and Supplementary Figure 2C, according to cluster 1 and 2.

Interestingly, the same patient clusters were obtained when analyzing the fold change of genes known to be involved in the EMT pathway from Gene Ontology (GO). Among these genes, Wnt pathway (*LEF1, TWIST1, SFRP2, WNT5A, DAB2*), TGFβ pathway (*TGFB1I1*, *TGFB3, DAB2*) and NOTCH1 pathway (*HEYL*) related genes, as well as *LOXL3* and *NFATC1*, showed significantly increased expression after cetuximab treatment in patients from cluster 1 (Figure 2H). *DAB2*, *LEF1* and *TWIST1* were found in both the EMT enriched and the EMT GO genes. Other genes implicated in the NOTCH1 pathway (*HAS2, HEY1, HEY2*), the WNT pathway (*WNT2* and *WNT4*), and the TGFβ pathway (*TGFBR3*), as well as *HGF*, showed increased expression (*P* < 0.1) after treatment in cluster 1 patients (Supplementary Figure 2D). In contrast, only few genes had expression modification in cluster 2 (Supplementary Figure 2E).

**Figure 3:**
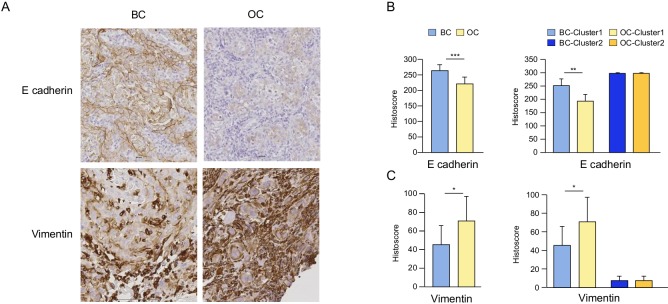
The impact of cetuximab treatment in SCCHN patients on E-cadherin and vimentin Representative immunohistochemical (IHC) staining of E-cadherin and vimentin on baseline (BC) and post-cetuximab (OC) tumor biopsies in a typical patient included in the group treated with cetuximab **A.**. Histoscore for E-cadherin **B.** and vimentin **B.** comparing BC and OC biopsies, as well as biopsies from patients from clusters 1 and 2 defined in Figure 2F. Histoscore = (% weakly stained cells) + (% moderately stained cells) × 2 + (% strongly stained cells) × 3.

To validate these results and further investigate EMT, additional analyses were performed by IHC. E-cadherin and vimentin were immunohistochemically stained on BC and OC biopsies of the cetuximab and control groups. We observed a significant decrease in E-cadherin expression and a significant increase in vimentin in tumor cells in the cetuximab group but not in the controls (data not shown), as semi-quantified by histoscore (Figure 3A). Patient clusters were also confirmed in this analysis; a decrease in E-cadherin and an increase in vimentin were found only in tumors from cluster 1 (Figure 3B, 3C).

### Cetuximab induced activation of CAFs

**Figure 4:**
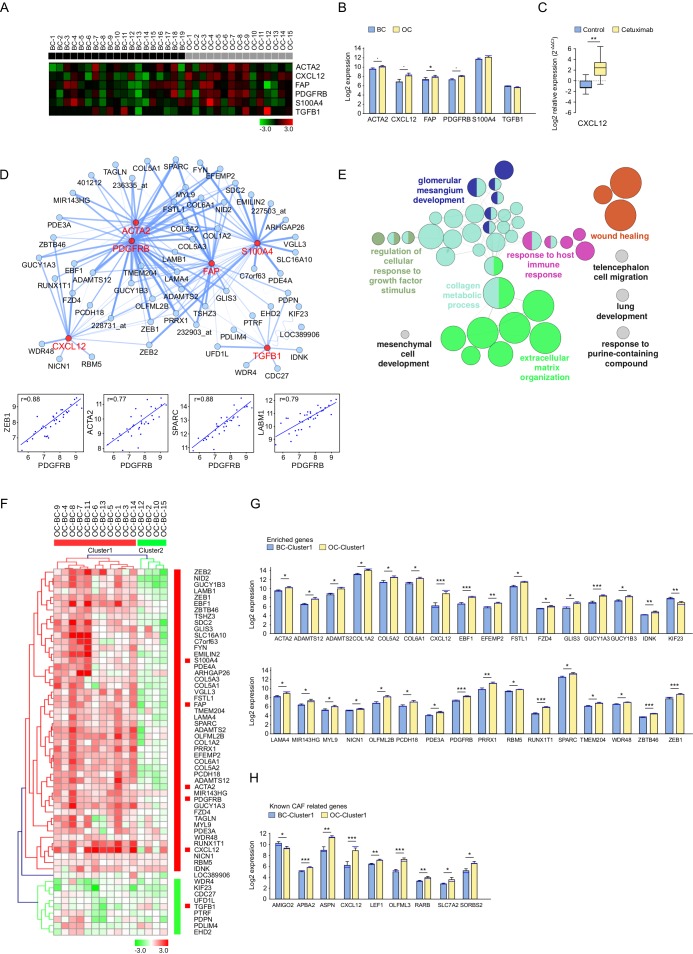
Expression pattern of cancer-associated fibroblast (CAF) markers and related genes after treatment with cetuximab in SCCHN patients **A.** The expression of known CAF related genes in baseline (BC, *n* = 19, black) and post-operative (OC, *n* = 15, grey) biopsies. Data was normalized. High and low expression is shown in red and green, respectively. **B.** CAF marker expression in OC (yellow) and BC (blue). Bar charts represent the mean (± SEM), and the median expression is shown in blue. Wilcoxon-Mann-Whitney test was applied (* 0.01 ≤ *P* < 0.05, 0.05 ≤ *P* < 0.1). **C.** RT-qPCR measured expression of CXCL12 in OC (yellow) and BC (blue). Whiskers represent means ± SD. **D.** CluePedia network showing CAF markers (red) together with correlating genes (blue). The edges represent Pearson r values (*P* > 0.6). Negative correlations are shown with a sinusoidal line. Correlation examples are shown for PDGFRB and enriched genes. **E.** ClueGO functional analysis of the CAF markers and enriched genes described at **D.**. Functionally grouped network with terms as nodes linked based on their kappa score. The node size represents the term enrichment significance. Fusion was applied to remove redundant terms. Only pathways with *P* < 0.05 after Benjamini correction for multiple testing were included in the network. Functionally related groups partially overlap. **F.** The fold change expression (OC-BC) of CAF markers and enriched genes described at **D.** in patients with complete time points (*n* = 15). The fold change matrix was normalized and hierarchical clustered (Kendall's tau, Average Linkage). Two patient clusters were revealed. Increased and decreased expressionis shown in red and green, respectively. Significantly increased expression after cetuximab treatment of **G.** CAF markers and enriched genes described at **D.**, as well as of **H.** genes known to be associated with cancer-associated fibroblasts^25^ in patients from cluster 1 **F.**. Bar charts represent the mean (± SEM) in OC (yellow) and BC (blue) and the median expression is shown in blue. Wilcoxon-Mann-Whitney test was applied (* 0.01 ≤ *P* < 0.05, ** 0.001 ≤ *P* < 0.01, ***P < 0.001). Genes with a similar trend can be seen in Supplementary Figure. 3.

To assess the impact of cetuximab on CAF-related genes, a methodology similar to that for EMT was applied. Six known CAF markers: *ACTA2, CXCL12, FAP, PDGFRB, S100A4* and *TGFB1* [18] were analyzed, and their expression was compared between BC and OC (Figure 4A, 4B). A significantly increased expression in OC was observed for *FAP*, with a similar trend for *ACTA2, CXCL12* and *PDGFRB*.

The expression of *CXCL12* was further investigated by qRT-PCR. A significantly upregulated *CXCL12* expression was observed in the cetuximab group but not in the controls, validating the microarray data (*P* = 0.004 respectively) (Figure 4C).

CAF enriched genes were further selected from the genes with the strongest correlation with the CAF markers, and were visualized together in a network (Figure 4D). Correlations between *PDGFRB* and other CAF-related genes were shown. Interestingly, *ZEB1* was part of both networks and correlated very well with many EMT and CAF related markers. Genes showing similar behaviour to the CAF markers after cetuximab treatment were involved in pathways relating to wound healing, the collagen metabolic process [21] and extracellular matrix organization (Figure 4E), as revealed by the ClueGO functional analysis.

Interestingly, the fold change of the CAF enriched gene expression observed in paired OC and BC samples identified the same patient clusters as obtained with the EMT enriched genes (Figure 4F, Supplementary Table 4). Statistically significant genes (*P* < 0.05) were represented in Figure 4G for cluster 1 and in Supplementary Figure 3A for all patients. Genes with a similar trend are shown in Supplementary Figure 3B and 3C, according to clusters 1 and 2.

Since GO does not yet provide a CAF-related term gene association, a previously published CAF gene signature defined in colon cancer was analysed [22]. Significantly differentially expressed genes are represented in Figure 4H, and gene expression modifications with a *P* < 0.1 are represented in Supplementary Figure 3D and Supplementary Figure 3E.

CAF enriched genes were further analysed together with a previously published stromal signature defined in breast cancer [23]. Many of the CAF and stroma-related genes were involved in the same biological functions (Supplementary Figure 3F).

Strong correlations between EMT and CAF enriched genes (r > 0.8) and markers (r > 0.6) were observed inside the tumor (Supplementary Figure 4A and 4B). Several genes, such as *ZEB1*, *ZEB2*, *FYN* and *LAMA4*, were found to be enriched in both EMT and CAF, suggesting similar functional implications.

To further validate both patient clusters, we tested 4 previously described SCCHN molecular subtypes in our microarray dataset [24]. Whereas most of the genes in the OC biopsy of cluster 1 were related to the mesenchymal phenotype, cluster 2 expressed genes related to the atypical phenotype after the treatment (Supplementary Figure 4C and 4D).

## DISCUSSION

This «window study» provided a unique opportunity to monitor the molecular modifications induced by cetuximab in treatment-naïve patients with SCCHN. Besides its antitumoral effect, we demonstrate here that cetuximab induces major tumor modifications. These modifications included genes and proteins implicated in the extracellular matrix, linked with CAFs or related to EMT.

We observed significant upregulation of decorin expression, which may play a role in tumor control, as suggested by the inverse correlations observed between decorin expression and the residual tumor cellularity of the surgical specimens. This small leucine-rich proteoglycan may inhibit tumor cell growth, migration, angiogenesis and metastasis [25, 26] by interaction with different receptor tyrosine kinases such as EGF [27, 28], other erbB family members [29], c-Met [30] and IGF-1R [31] and upregulation of p21WAF1/CIP1 (p21) via a p-53 independent pathway [32]. SPARCL1 (hevin), another matricellular protein was also found to be upregulated in our cetuximab treated tumors. This protein seems to modulate the structure of the extracellular matrix by regulating decorin levels and collagen fibril assembly [33].

Besides an increased expression of matrikines, which may play a role in the control of tumor growth and decreased tumor invasion, we observed tumor modifications that may lead to tumor regrowth, or have the potential to be implicated in cetuximab resistance. We showed extracellular matrix modifications of the tumor and upregulation of factors generally secreted by CAFs such as CXCL12, ASPN [34] and OLFML3 [35, 36]. CAFs may induce resistance to cetuximab [12]. Cetuximab-induced growth inhibition was reduced in SCCHN cell lines co-cultured with CAFs through CAF-derived soluble factors, and CAF induced resistance to cetuximab was partly abolished by matrix metalloproteinase inhibitors.

The upregulation of CXCL12 is of particular interest. CXCL12, also known as stromal cell-derived factor-1α, is the ligand of CXCR4. Activation of the CXCR4/CXCL12 axis can lead to tumor proliferation and can promote neoangiogenesis, cancer cell invasion and metastasis, and EMT [37, 38]. Moreover, CXCR4 may have a role in cancer stem cells in SCCHN [39]. It is also expressed on circulating tumor cells of metastatic carcinoma and in melanoma patients [40]. Inhibitors of the CXCR4/CXCL12 axis are currently under development [41].

Furthermore, we showed high correlations between the known CAF markers and other genes potentially implicated in EMT, including a high correlation between *PDGFRB* and *LAMB1*, and *PDGFR* and *ZEB1*. It has been described that PDGF is required for the translation of *LAMB1* during EMT [42].

Finally, increased EMT has been shown to be a potential mechanism of anti-EGFR resistance in pre-clinical models [11, 43, 44]. We investigated the expression of different EMT markers and showed that some patients have upregulation of EMT markers after cetuximab. We identified two groups with different EMT characteristics. Whereas cluster 1 patients showed clear upregulation of expression of multiple genes implicated in EMT and embryologic pathways like NOTCH and Wnt, cluster 2 patients did not show the same modifications in gene expression. These findings may have therapeutic implications since the addition of Wnt inhibitors to EGFR inhibitors showed increased activity [45, 46] in non-small cell lung carcinoma cells (NSCLC). Our study showed increased expression of markers implicated in the noncanonical frizzled2 pathway (*SFRP2, WNT5A, FYN)*, which has been recently identified by Gujral et al. [47]. Inhibition of this pathway, implicated in EMT and cell migration, shows encouraging results on xenografts. It is currently unknown whether cetuximab enriches mesenchymal-like cells or promotes the transformation of epithelial to mesenchymal cell types by EMT. The correlation between DCN and EMT markers plays in favor of the first hypothesis.

One limitation of our work is that only three doses of cetuximab were given to avoid delaying standard surgical curative treatment for ethical reasons. Our model can therefore only investigate early occurring molecular mechanisms. Other designs are needed to fully explore the long-term acquired resistance mechanisms of anti-EGFR monoclonal antibodies. Other limitations are the low number of patients, a possible bias due to tumor heterogeneity as well as the difficulties to perform more precise protein validation of the gene expression profiles due to the limited amount of tumor tissues we can collect safely in a real clinical situation.

Our previous work and this study collectively demonstrate that cetuximab reduces tumor cellularity and induces modifications in the peritumoral microenvironment of SCCHN. Even if EMT and CAFs have shown to be possibly implicated in cetuximab resistance in pre-clinical models, our data show that these molecular processes may also occur very early on in the clinic. Our work paves the way for further investigation of novel therapies targeting these pathways in SCCHN.

## MATERIALS AND METHODS

### Patients

Cetuximab was administered for two weeks prior to surgery to 33 treatment-naïve patients (NCT00714649). Five control patients were included. They were not treated with cetuximab but were subject to the same biopsy and imaging requirements. Details of the eligibility criteria, pretreatment evaluation, safety, and clinical results have been published [16]. The clinical and translational parts of the study were approved by the Independent Ethics Committee and the Belgian Health Authorities and conducted in accordance with the Declaration of Helsinki (October 2000). It was prospectively planned to perform translational research and patients gave their informed consent for repeated biopsies. For homogeneity, only the patients included in the expansion part of the trial (*n* = 20) and the control patients (*n* = 5) were included in the translational research reported here.

### Tissue samples

Tumor biopsies were taken from the middle of the tumor surface at two different time points: (i) before treatment, baseline condition samples (BC), and (ii) on the day of surgery, operation condition samples (OC). At each time point, one biopsy was stored in RNAlater^®^ for up to one week at 4°C and then frozen at −80°C, and another was fixed in 4% formalin and embedded in paraffin.

### Immunohistochemistry and pathologic evaluation

Slides coming from tumor biopsies and the resected tumor were haematoxylin and eosin (H&E) stained to confirm the presence of viable tumor cells.

Immunohistochemical (IHC) staining was performed on 4-μm paraffin embedded tumor sections for p21WAF (cell signaling, 1/75), E-cadherin (cell signaling, 1/100) and vimentin (cell signaling, 1/100), and on surgical specimens for decorin (R&D, 1/40). All the stained slides were digitized by a slide scanner (Mirax Scan; Zeiss, Jena, Germany). The percentage of the stained area was evaluated using Frida software (http://bui3.win.ad.jhu.edu/frida). p21 and decorin expression were subsequently quantified using an optical microscope at 400x and 200x magnification, respectively. E-cadherin and vimentin staining on tumor cells was blindly assessed for each patient by histoscore as previously published [16].

### RNA extraction and microarrays

Total RNA was extracted using the miRNeasy Mini Kit from Qiagen (Venlo, Netherlands).

After checking the concentration (NanoDrop), yield and integrity (Agilent 2100 Bioanalyzer), RNA samples of high quality (RNA integrity number (RIN) > 6, mean 8.1) were selected for gene expression profiling by microarray. According to these criteria, one BC and five OC biopsies were not analyzed by microarray. 250 ng of RNA was amplified and labeled using the Affymetrix GeneChip^®^ 3′ IVT Express Kit. RNA was hybridized overnight to Affymetrix GeneChip HG-U133 Plus 2.0 (High Wycombe, UK), then stained and washed on an Affymetrix Gene Chip^®^ Fluidics Station 450. The array was then scanned to produce an image data file (DAT) and a cell intensities data file (CEL). A report file (RPT) was generated and a chip data file (CHP) was generated from the CEL file. The CEL file was then exported, ready for importing the analysis.

Based on the bioinformatic analysis quality control procedure, no microarrays were removed. The dataset was normalized using the GCRMA method. Normalized expression levels are given in Log2 scale.

### Reverse transcriptase polymerase chain reaction (RT-PCR) and real time quantitative RT-PCR (qRT-PCR)

Synthesis of first-strand cDNA for use in qRT-PCR was carried out on 500 ng of total mRNA and subjected to reverse transcription using SuperScript^®^ III First-Strand Synthesis SuperMix for qRT-PCR, as described by the manufacturer (Invitrogen).

The following predesigned TaqMan^®^ gene expression assays and TaqMan^®^ Gene Expression Master Mix were obtained from AB Applied Biosystems:

DCN (Hs00370384_m1), CXCL12 (Hs00171022_m1), ZEB1 (Hs00232783_m1), OLFML3 (Hs00220180_m1), p21 (Hs00355782_m1), TWIST1 (Hs00361186_m1), TWIST2 (Hs00382379_m1), LEF1 (Hs01547250_m1).

Human glyceraldehyde 3 phosphate dehydrogenase (GAPDH) (AB Applied Biosystems NM_002046.3) was used as an internal control as previously described (20). qRT-PCR was performed in a 10μl total reaction mixture using a CFX96™ (Bio-Rad) thermal cycler. The thermal cycling conditions were 50°C for two minutes followed by 95°C for 10 minutes, 95°C for 15 seconds and 60°C for 60 seconds. The last two steps were repeated for 40 cycles. Real-time PCR was performed in duplicate for each gene.

The qRT-PCR data was presented as delta Ct values of the investigated genes relative to GADPH.

The fold change (2-ΔΔCt) in gene expression in each paired sample was calculated using the formulas: gene expression ΔCt = average gene expression Ct minus average GADPH Ct; gene expression ΔΔCt = gene expression ΔCt OC minus gene expression ΔCt BC. Results were expressed in Log2 scale.

### Statistical methods

The normality of the data was tested using the Shapiro-Wilk test. The Student's t-test and the Mann-Whitney-Wilcoxon rank-sum test were used for pairwise comparisons of parametric and non-parametric data, respectively. Pearson and Wilcoxon correlations were performed. A *P* - value < 0.05 was considered statistically significant. Benjamini-Hochberg method was used for multiple testing correction.

Genes were mapped on Gene Ontology (GO), Kyoto Encyclopedia of Genes and Genomes (KEGG) and Reactome (data was downloaded on 01.02.2014) and enriched (*P* < 0.05 after Bonferroni step-down correction), non redundant pathways and terms were visualized in ClueGO networks [17].

Using CluePedia [20], correlations between the expression of epithelial to mesenchymal transition (EMT) and cancer-associated fibroblast (CAF) markers and other Affymetrix tested genes, were calculated. Genes with the highest correlation were extracted and enriched the EMT and CAF networks, respectively. The expression difference in the OC compared to the BC was calculated for the EMT and CAF markers and for the enriched genes. The expression difference matrix was normalized and clustered in Genesis (Kendall's tau distance measurement, Average Linkage).

## SUPPLEMENTARY MATERIAL FIGURES AND TABLES


