# Supplementary material for: Cetuximab promotes epithelial to mesenchymal transition and cancer associated fibroblasts in patients with head and neck cancer
Source: Oncotarget. 2015 Sep 30;6(33):34288–99. doi: 10.18632/oncotarget.5924 (PMC4741452; doi:10.18632/oncotarget.5924)
Supplement: Supplementary file 1 [file oncotarget-06-34288-s001.pdf]

Cetuximab promotes epithelial to mesenchymal transition and cancer associated fibroblasts in patients with head and neck cancer

Supplementary Material

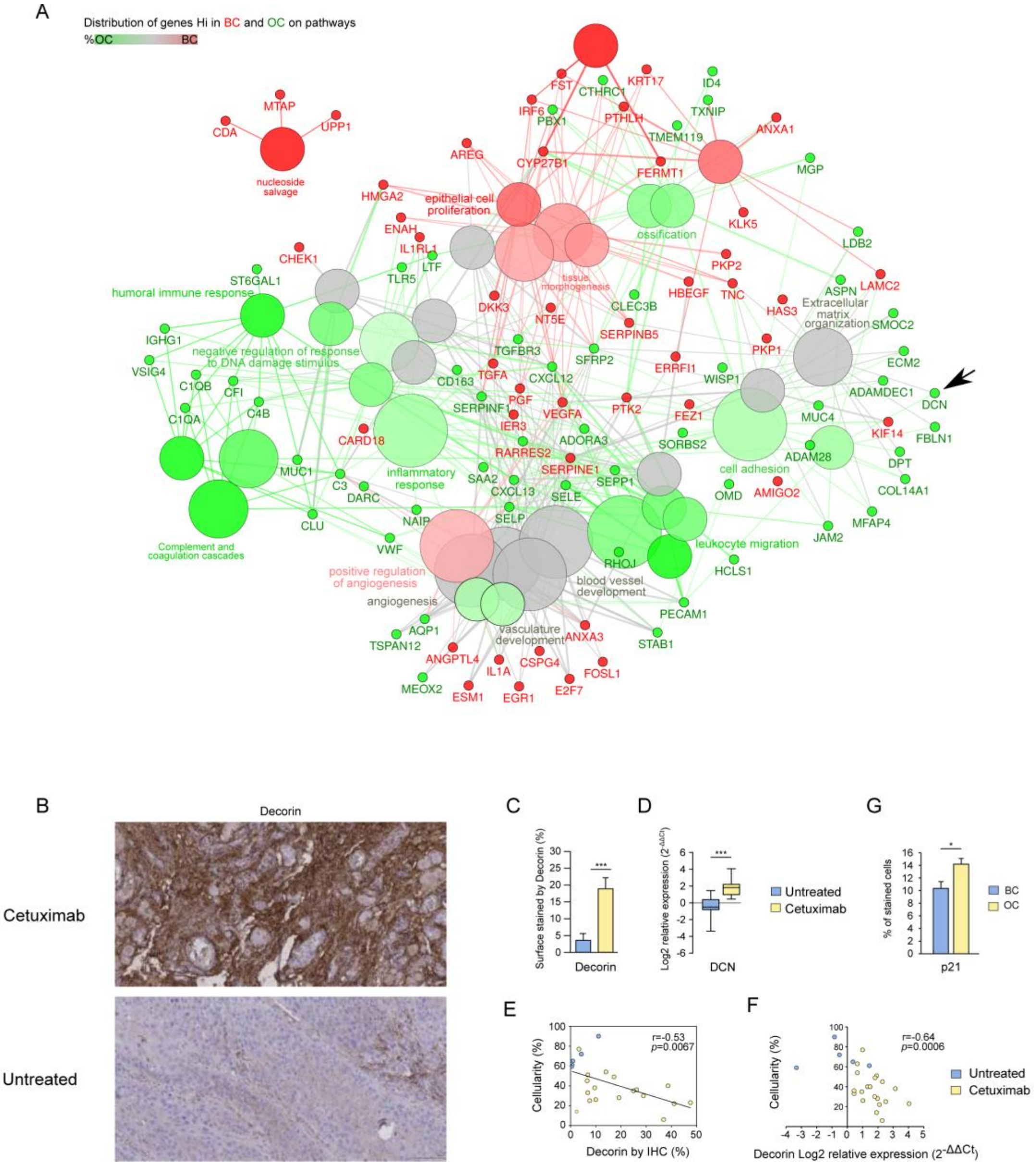

Figure S1: Biological role of the differently expressed genes between the pre and post-treatment biopsies.

A. Pathways and terms described in Figure 1C and their associated genes with high expression in BC (red) and OC (green). Terms having predominantly associated high expression genes in BC

and OC are shown in red and green, respectively. The colour gradient shows the gene proportion of the genes with high expression in BC and OC associated with the term. Equal proportion is represented in grey.

B. Representative immunohistochemical (IHC) staining of decorin in the operative specimen of a patient treated with cetuximab and in a untreated patient.

C. Percentage of surface stained with decorin in surgical specimens in cetuximab treated (yellow) and untreated patients (blue), *** $P < 0.001$

D. Relative RNA expression levels (RT-qPCR) of decorin (DCN) in cetuximab treated (OC, yellow) and not treated (BC, blue) biopsies. Whiskers represent means ± SD (standard deviation). *** $P < 0.001$

E. Pearson correlation between tumour cellularity (%) and % of surface stained by decorin by IHC on slides of surgically resected specimens treated with cetuximab. Tumour cellularity (expressed as a percentage) was the surface occupied by tumour cells divided by the surface of the whole tumour that included tumour cells, inflammatory cells, normal interstitial tissue and areas with morphologic signs of therapy-induced regression such as fibrosis and scarring.

F. Pearson correlation between tumour cellularity (%) and decorin expression measured by qPCR.

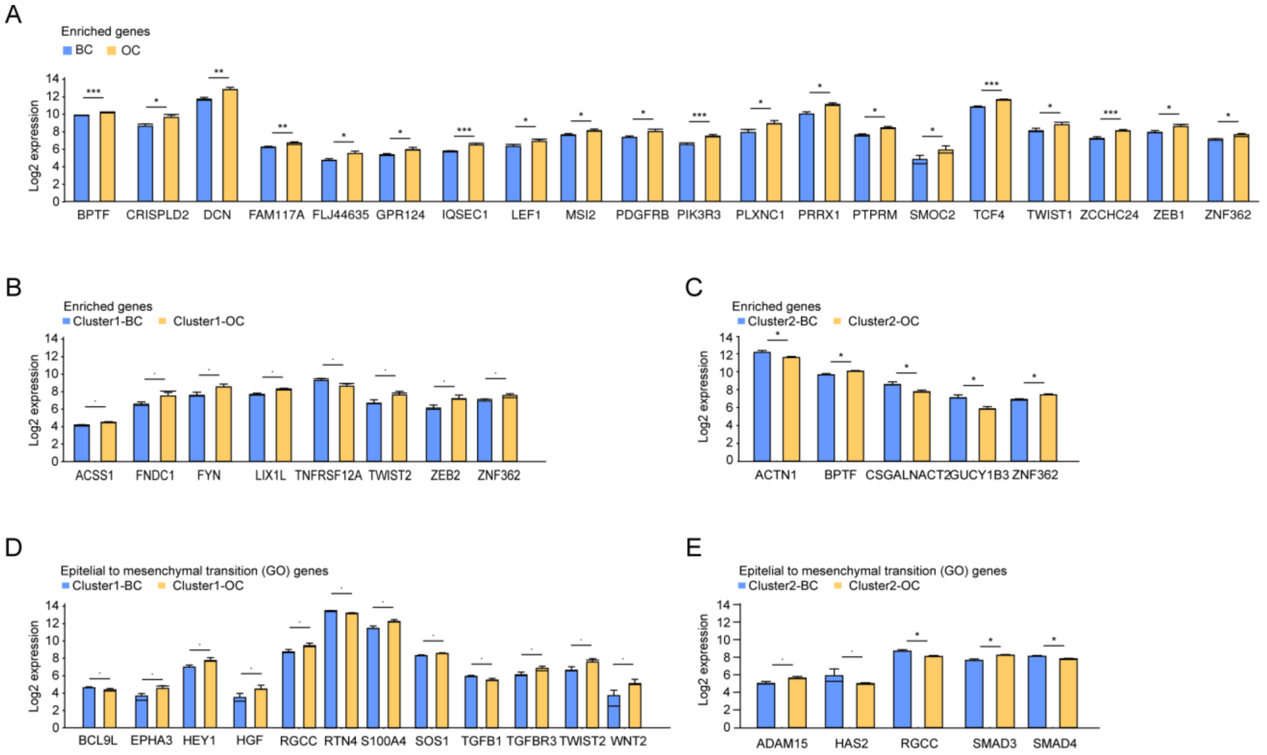

Figure S2: Different expression pattern of epithelial to mesenchymal transition (EMT) markers and related genes after treatment with cetuximab in SCCHN patients.

Affymetrix measured EMT marker expression in OC (yellow) and BC (blue) in all samples (BC, n=19, OC, n=15) (A), or in patients with complete time points included in cluster 1 (B, D) or cluster 2 (C, E), defined in Figure 2F. Bar charts represent the mean (± SEM) and the median expression is shown in blue. Statistical analyses were performed by the Wilcoxon–Mann–Whitney method (. $0.05 \leq P < 0.1$, * $0.01 \leq P < 0.05$, ** $0.001 \leq P < 0.01$, *** $P < 0.001$).

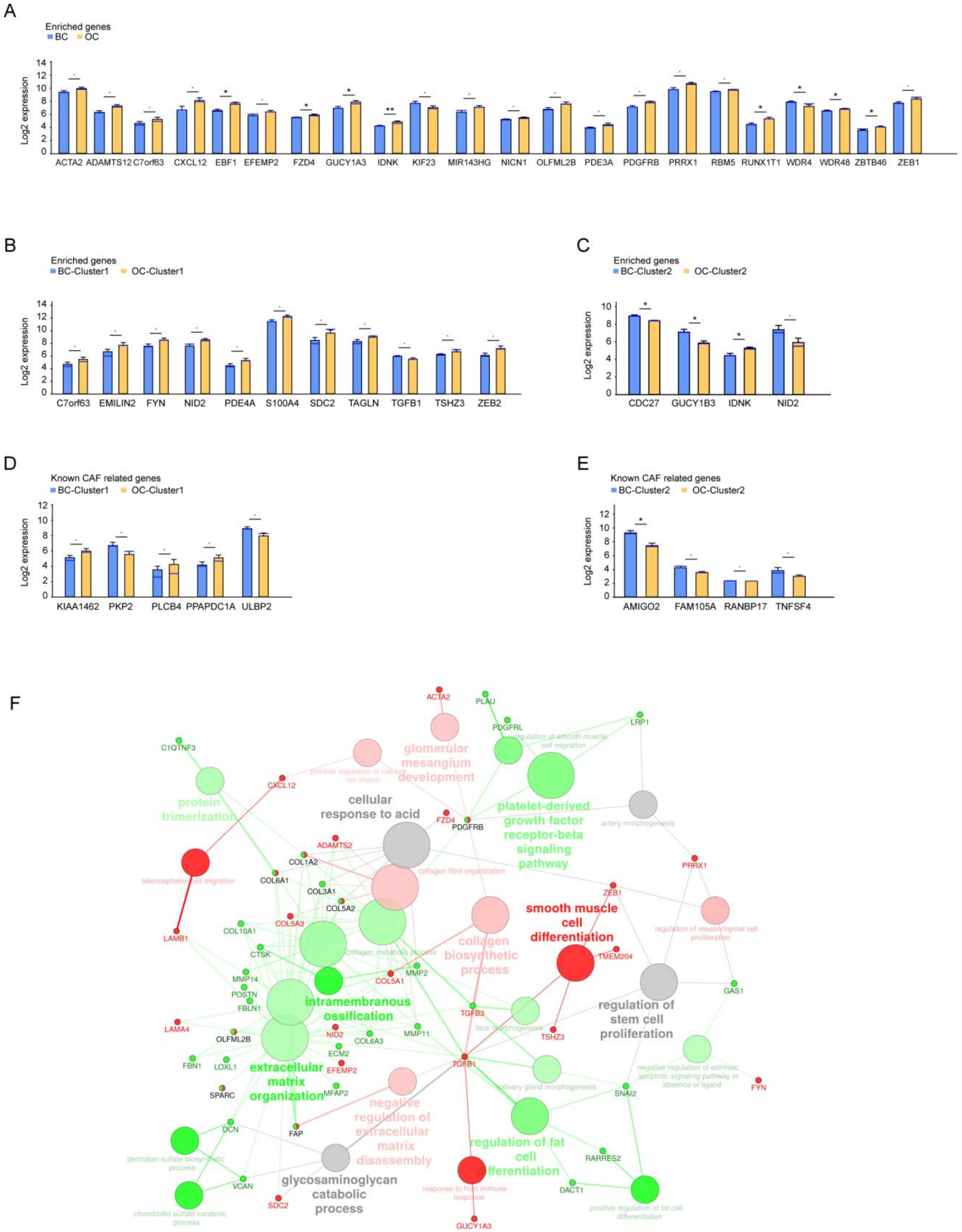

Figure S3: Different expression pattern of cancer-associated fibroblast (CAF) markers and related genes after treatment with cetuximab in SCCHN patients.

Affymetrix measured CAF marker expression in OC (yellow) and BC (blue) in all samples (BC, n=19, OC, n=15) (A), or in patients with complete time points included in cluster 1 (B, D) or cluster 2 (C, E), defined in Figure 4F. Bar charts represent the mean (± SEM) and the median expression is shown in blue. Statistical analyses were performed by the Wilcoxon–Mann–Whitney method (. $0.05 \leq P < 0.1$, * $0.01 \leq P < 0.05$, ** $0.001 \leq P < 0.01$, *** $P < 0.001$).

F. ClueGO functional analysis of CAF markers and enriched genes described in Figure 3F (red) and stroma related genes (green). Pathways and terms and their associated genes are visualized in a network. Terms having predominantly associated CAF markers and related genes, and stroma related genes, are shown in red and green, respectively. The colour gradient shows the gene proportion of the CAF or stroma genes associated with the term. Equal proportion is represented in grey.

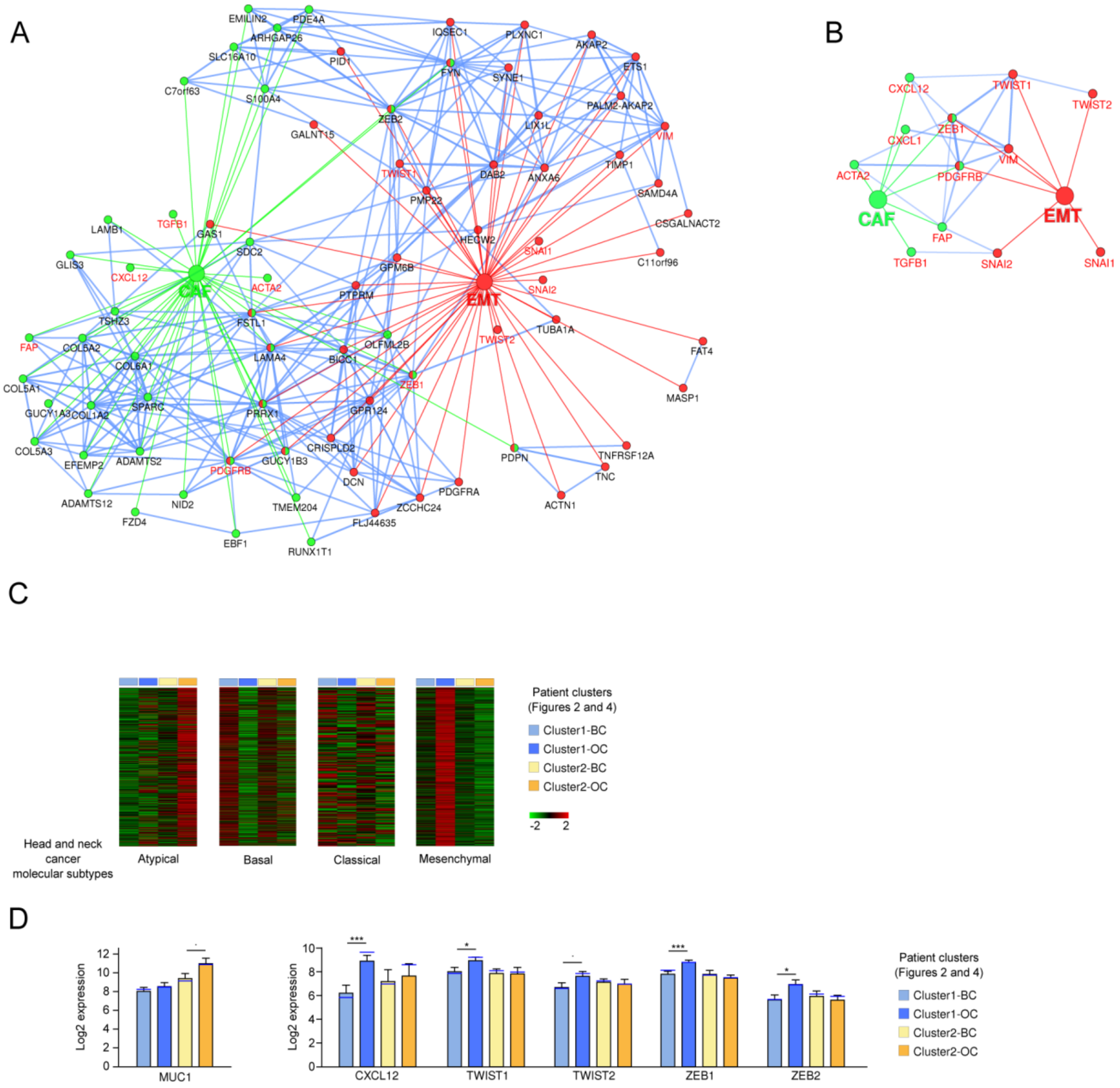

Figure S4: EMT and CAF markers and enriched genes correlate in human head and neck cancer.

EMT and CAF enriched gene (A) and known marker (B) expression correlation visualized in a ClueGO-CluePedia network. EMT and CAF markers are coloured in red and green, respectively. Pearson correlation values (A: r > 0.8, B: r > 0.6) are shown in blue. The Organic algorithm was applied to organize the network.

Table S1: Details regarding the 284 Affymetrix spots significantly differentially expressed ($P < 0.05$) between BC and OC.

High in	Affymetrix ID	Entrez Gene ID	Representative ID	Symbol	Name
BC	230778_at	---	AA010315	---	Transcribed locus
BC	208002_s_at	11332	NM_007274	ACOT7	acyl-CoA thioesterase 7
BC	222108_at	347902	AC004010	AMIGO2	adhesion molecule with Ig-like domain 2
BC	221009_s_at	51129	NM_016109	ANGPTL4	angiopoietin-like 4
BC	239196_at	118932	AI097229	ANKRD22	ankyrin repeat domain 22
BC	238439_at	118932	AI925518	ANKRD22	ankyrin repeat domain 22
BC	227337_at	353322	AA886870	ANKRD37	ankyrin repeat domain 37
BC	233011_at	301	AU155094	ANXA1	Annexin A1
BC	209369_at	306	M63310	ANXA3	annexin A3
BC	205239_at	374	NM_001657	AREG	amphiregulin (schwannoma-derived growth factor)
BC	203946_s_at	384	U75667	ARG2	arginase, type II
BC	228068_at	55118	AI563972	C10orf132	chromosome 10 open reading frame 132
BC	1555786_s_at	55673	BC008034	C14orf34	chromosome 14 open reading frame 34
BC	60474_at	55612	AA469071	C20orf42	chromosome 20 open reading frame 42
BC	229070_at	84830	AA470369	C6orf105	chromosome 6 open reading frame 105
BC	220513_at	80759	NM_030568	C6orf148	chromosome 6 open reading frame 148
BC	205627_at	978	NM_001785	CDA	cytidine deaminase
BC	228868_x_at	81620	AW075105	CDT1	DNA replication factor
BC	205393_s_at	1111	NM_001274	CHEK1	CHK1 checkpoint homolog (S. pombe)
BC	205394_at	1111	NM_001274	CHEK1	CHK1 checkpoint homolog (S. pombe)
BC	214297_at	1464	BE857703	CSPG4	Chondroitin sulfate proteoglycan 4 (melanoma-associated)
BC	220230_s_at	51700	NM_016229	CYB5R2	cytochrome b5 reductase 2
BC	205676_at	1594	NM_000785	CYP27B1	cytochrome P450, family 27, subfamily B, polypeptide 1
BC	230508_at	27122	AL569601	DKK3	Dickkopf homolog 3 (Xenopus laevis)
BC	228033_at	144455	AI341146	E2F7	E2F transcription factor 7
BC	231192_at	23566	AW274018	EDG7	Endothelial differentiation, lysophosphatidic acid G-protein-coupled receptor, 7
BC	220816_at	23566	NM_012152	EDG7	endothelial differentiation, lysophosphatidic acid G-protein-coupled receptor, 7
BC	201693_s_at	1958	AV733950	EGR1	early growth response 1
BC	235296_at	56648	BG500474	EIF5A2	eukaryotic translation initiation factor 5A2
BC	228260_at	1993	AL161628	ELAVL2	ELAV (embryonic lethal, abnormal vision, Drosophila)-like 2 (Hu antigen B)
BC	1553672_at	55740	NM_145240	ENAH	enabled homolog (Drosophila)
BC	235419_at	54206	AW612461	ERRFI1	ERBB receptor feedback inhibitor 1
BC	208394_x_at	11082	NM_007036	ESM1	endothelial cell-specific molecule 1
BC	238460_at	84985	AI590662	FAM83A	family with sequence similarity 83, member A
BC	203562_at	9638	NM_005103	FEZ1	fasciculation and elongation protein zeta 1 (zygin I)
BC	205014_at	9982	NM_005130	FGFBP1	fibroblast growth factor binding protein 1
BC	220724_at	80157	NM_025087	FLJ21511	hypothetical protein FLJ21511
BC	204420_at	8061	BG251266	FOSL1	FOS-like antigen 1
BC	204948_s_at	10468	NM_013409	FST	follistatin
BC	207345_at	10468	NM_006350	FST	follistatin
BC	226847_at	10468	BF438173	FST	follistatin
BC	223079_s_at	2744	AI828035	GLS	glutaminase
BC	221902_at	387509	AL567940	GPR153	G protein-coupled receptor 153
BC	64942_at	387509	AI937160	GPR153	G protein-coupled receptor 153
BC	232290_at	9052	BE815259	GPRC5A	G protein-coupled receptor, family C, group 5, member A
BC	223541_at	3038	AF232772	HAS3	hyaluronan synthase 3
BC	203821_at	1839	NM_001945	HBEGF	heparin-binding EGF-like growth factor
BC	38037_at	1839	M60278	HBEGF	heparin-binding EGF-like growth factor
BC	206685_at	54435	NM_018985	HCG4	HLA complex group 4
BC	1554452_a_at	29923	BC001863	HIG2	hypoxia-inducible protein 2
BC	218507_at	29923	NM_013332	HIG2	hypoxia-inducible protein 2
BC	208025_s_at	8091	NM_003483	HMGA2	high mobility group AT-hook 2 /// high mobility group AT-hook 2
BC	1567224_at	8091	U29113	HMGA2	high mobility group AT-hook 2
BC	205829_at	3292	NM_000413	HSD17B1	hydroxysteroid (17-beta) dehydrogenase 1
BC	231733_at	59082	NM_021571	ICEBERG	ICEBERG caspase-1 inhibitor
BC	201631_s_at	8870	NM_003897	IER3	immediate early response 3
BC	231148_at	147920	AI806131	IGFL2	insulin growth factor-like family member 2
BC	210118_s_at	3552	M15329	IL1A	interleukin 1, alpha
BC	207526_s_at	9173	NM_003856	IL1RL1	interleukin 1 receptor-like 1
BC	205376_at	8821	NM_003866	INPP4B	inositol polyphosphate-4-phosphatase, type II, 105kDa
BC	1552477_a_at	3664	BC014852	IRF6	interferon regulatory factor 6
BC	233002_at	57718	AB046842	KIAA1622	KIAA1622
BC	236641_at	9928	AW183154	KIF14	kinesin family member 14
BC	239853_at	147700	AI279514	KLC3	kinesin light chain 3
BC	222242_s_at	25818	AF243527	KLK5	kallikrein 5
BC	233687_s_at	284366	AC011473	KLK8	kallikrein 8 (neuropsin/ovasin)
BC	205157_s_at	3872	NM_000422	KRT17	keratin 17
BC	212236_x_at	3872	Z19574	KRT17	keratin 17
BC	213680_at	3854	AI831452	KRT6B	keratin 6B
BC	207517_at	3918	NM_018891	LAMC2	laminin, gamma 2
BC	1554097_a_at	554202	BC021861	LOC554202	hypothetical LOC554202
BC	1557285_at	374	AI891075	LOC653193	Similar to Amphiregulin precursor (AR) (Colorectum cell-derived growth factor) (CRDGF)
BC	236039_at	284348	N31975	LYPD5	LY6/PLAUR domain containing 5
BC	220651_s_at	55388	NM_018518	MCM10	MCM10 minichromosome maintenance deficient 10 (S. cerevisiae)
BC	211363_s_at	4507	AF109294	MTAP	methylthioadenosine phosphorylase
BC	1552658_a_at	89795	NM_014903	NAV3	neuron navigator 3
BC	204823_at	89795	NM_014903	NAV3	neuron navigator 3
BC	240991_at	10397	AI732596	NDRG1	N-myc downstream regulated gene 1
BC	203939_at	4907	NM_002526	NT5E	5'-nucleotidase, ecto (CD73)
BC	1553995_a_at	4907	BC015940	NT5E	5'-nucleotidase, ecto (CD73)
BC	1553994_at	4907	BC015940	NT5E	5'-nucleotidase, ecto (CD73)
BC	225314_at	132299	BG291649	OCIAD2	OCIA domain containing 2
BC	242871_at	54852	AI934557	PAQR5	progestin and adipoQ receptor family member V
BC	209652_s_at	5228	BC001422	PGF	placental growth factor, vascular endothelial growth factor-related protein
BC	205724_at	5317	NM_000299	PKP1	plakophilin 1 (ectodermal dysplasia/skin fragility syndrome)
BC	207717_s_at	5318	NM_004572	PKP2	plakophilin 2
BC	219702_at	10761	NM_021796	PLAC1	placenta-specific 1
BC	218644_at	26499	NM_016445	PLEK2	pleckstrin 2
BC	211756_at	5744	BC005961	PTHLH	parathyroid hormone-like hormone /// parathyroid hormone-like hormone
BC	210355_at	5744	J03580	PTHLH	parathyroid hormone-like hormone
BC	206300_s_at	5744	NM_002820	PTHLH	parathyroid hormone-like hormone
BC	241453_at	5747	AA912743	PTK2	PTK2 protein tyrosine kinase 2
BC	219412_at	23682	NM_022337	RAB38	RAB38, member RAS oncogene family
BC	203423_at	5947	NM_002899	RBP1	retinol binding protein 1, cellular
BC	210138_at	8601	AF074979	RGS20	regulator of G-protein signalling 20
BC	209885_at	29984	BC001338	RHOD	ras homolog gene family, member D
BC	31846_at	29984	AW003733	RHOD	ras homolog gene family, member D
BC	227072_at	25914	BG167480	RTTN	rotatin
BC	204268_at	6273	NM_005978	S100A2	S100 calcium binding protein A2
BC	1555551_at	5268	BC020713	SERPINB5	serpin peptidase inhibitor, clade B (ovalbumin), member 5
BC	1568765_at	5054	BC020765	SERPINE1	serpin peptidase inhibitor, clade E (nexin, plasminogen activator inhibitor type 1), member 1
BC	230973_at	400745	AI937119	SH2D5	SH2 domain containing 5
BC	206628_at	6523	NM_000343	SLC5A1	solute carrier family 5 (sodium/glucose cotransporter), member 1
BC	221489_s_at	81848	W48843	SPRY4	sprouty homolog 4 (Drosophila)
BC	205016_at	7039	NM_003236	TGFA	transforming growth factor, alpha
BC	205015_s_at	7039	M31172	TGFA	transforming growth factor, alpha
BC	215033_at	4071	AI189753	TM4SF1	transmembrane 4 L six family member 1
BC	222892_s_at	55287	AI087937	TMEM40	transmembrane protein 40
BC	219503_s_at	55287	NM_018306	TMEM40	transmembrane protein 40
BC	241272_at	3371	BE156417	TNC	Tenascin C (hexabrachion)
BC	203234_at	7378	NM_003364	UPP1	uridine phosphorylase 1
BC	211527_x_at	7422	M27281	VEGF	vascular endothelial growth factor

BC	203798_s_at	7447	NM_003385	VSNL1	visinin-like 1
BC	219836_at	79413	NM_024508	ZBED2	zinc finger, BED-type containing 2
OC	1556185_a_at	---	BC035072	---	CDNA clone IMAGE:5260162
OC	235427_at	---	AA418074	---	Transcribed locus
OC	236045_x_at	441801	AW451197	---	CDNA clone IMAGE:5278089
OC	227061_at	---	AI088063	---	CDNA FLJ44429 fis, clone UTERU2015653
OC	236220_at	---	AI935541	---	Transcribed locus
OC	229802_at	8840	AA147884	---	CDNA FLJ14388 fis, clone HEMBA1002716
OC	235122_at	---	AI800713	---	CDNA clone IMAGE:6254031
OC	226252_at	---	AA058578	---	CDNA FLJ34585 fis, clone KIDNE2008758
OC	217227_x_at	---	X93006	---	IgG lambda light chain V-J-C region (clone Tgl11)
OC	226625_at	7049	AW193698	---	---
OC	227719_at	---	AA934610	---	Transcribed locus
OC	228827_at	862	AI217416	---	MRNA; cDNA DKFZp564B213 (from clone DKFZp564B213)
OC	228528_at	---	AI927692	---	CDNA FLJ41270 fis, clone BRAMY2036387
OC	227082_at	---	AI760356	---	MRNA; cDNA DKFZp586K1922 (from clone DKFZp586K1922)
OC	238668_at	---	AI130690	---	Transcribed locus, strongly similar to NP_848718.1 mitochondrial ribosomal protein L50 [Mus musculus]
OC	227646_at	1879	BG435302	---	CDNA FLJ39389 fis, clone PLACE6003621
OC	230391_at	---	BF439449	---	---
OC	204719_at	10351	NM_007168	ABCA8	ATP-binding cassette, sub-family A (ABC1), member 8
OC	226030_at	36	BE897866	ACADSB	acyl-Coenzyme A dehydrogenase, short/branched chain
OC	205997_at	10863	NM_021778	ADAM28	ADAM metallopeptidase domain 28
OC	206134_at	27299	NM_014479	ADAMDEC1	ADAM-like, decysin 1
OC	230800_at	196883	AV699353	ADCY4	adenylate cyclase 4
OC	223660_at	140	AF226731	ADORA3	adenosine A3 receptor
OC	213592_at	187	X89271	AGTRL1	angiotensin II receptor-like 1
OC	202920_at	287	BF726212	ANK2	ankyrin 2, neuronal
OC	1569607_s_at	440841	BC016022	ANKRD20A1	ankyrin repeat domain 20 family, member A1
OC	230925_at	54518	AI093231	APBB1IP	amyloid beta (A4) precursor protein-binding, family B, member 1 interacting protein
OC	204416_x_at	341	NM_001645	APOC1	apolipoprotein C-I
OC	207542_s_at	358	NM_000385	AQP1	aquaporin 1 (Colton blood group)
OC	209047_at	358	AL518391	AQP1	aquaporin 1 (Colton blood group)
OC	244061_at	55843	AI510829	ARHGAP15	Rho GTPase activating protein 15
OC	224396_s_at	54829	AF316824	ASPN	asporin (LRR class 1) /// asporin (LRR class 1)
OC	219087_at	54829	NM_017680	ASPN	asporin (LRR class 1)
OC	213036_x_at	489	Y15724	ATP2A3	ATPase, Ca++ transporting, ubiquitous
OC	207522_s_at	489	NM_005173	ATP2A3	ATPase, Ca++ transporting, ubiquitous
OC	229127_at	522	BF195118	ATP5J	ATP synthase, H+ transporting, mitochondrial F0 complex, subunit F6
OC	204860_s_at	4671	AI817801	BIRC1	baculoviral IAP repeat-containing 1
OC	233036_at	54796	AU146418	BNC2	Basonuclin 2
OC	212771_at	221061	AU150943	C10orf38	chromosome 10 open reading frame 38
OC	218546_at	79762	NM_024709	C1orf115	chromosome 1 open reading frame 115
OC	218232_at	712	NM_015991	C1QA	complement component 1, q subcomponent, A chain
OC	202953_at	713	NM_000491	C1QB	complement component 1, q subcomponent, B chain
OC	217767_at	718	NM_000064	C3	complement component 3
OC	208451_s_at	721	NM_000592	C4A	complement component 4A (Rodgers blood group)
OC	212914_at	23492	AV648364	CBX7	chromobox homolog 7
OC	205392_s_at	6358	NM_004166	CCL14	chemokine (C-C motif) ligand 14
OC	221511_x_at	9236	AB033080	CCPG1	cell cycle progression 1
OC	215049_x_at	9332	Z22969	CD163	CD163 molecule
OC	203645_s_at	9332	NM_004244	CD163	CD163 molecule
OC	203799_at	9936	NM_014880	CD302	CD302 molecule
OC	203854_at	3426	NM_000204	CFI	complement factor I
OC	213060_s_at	1117	U58515	CHI3L2	chitinase 3-like 2 /// chitinase 3-like 2
OC	205200_at	7123	NM_003278	CLEC3B	C-type lectin domain family 3, member B
OC	227742_at	54102	AI638295	CLIC6	chloride intracellular channel 6
OC	208791_at	1191	M25915	CLU	clusterin
OC	208792_s_at	1191	M25915	CLU	clusterin
OC	238106_at	---	AW771190	COBLL1	COBL-like 1
OC	228750_at	---	AI693516	COL14A1	Collagen, type XIV, alpha 1 (undulin)
OC	212865_s_at	7373	BF449063	COL14A1	collagen, type XIV, alpha 1 (undulin)
OC	225681_at	115908	AA584310	CTHRC1	collagen triple helix repeat containing 1
OC	203666_at	6387	NM_000609	CXCL12	chemokine (C-X-C motif) ligand 12 (stromal cell-derived factor 1)
OC	209687_at	6387	U19495	CXCL12	chemokine (C-X-C motif) ligand 12 (stromal cell-derived factor 1)
OC	205242_at	10563	NM_006419	CXCL13	chemokine (C-X-C motif) ligand 13 (B-cell chemoattractant)
OC	222453_at	79901	AL136693	CYBRD1	cytochrome b reductase 1
OC	227702_at	260293	AA557324	CYP4X1	cytochrome P450, family 4, subfamily X, polypeptide 1
OC	208335_s_at	2532	NM_002036	DARC	Duffy blood group, chemokine receptor
OC	209335_at	1634	AI281593	DCN	decorin
OC	211896_s_at	1634	AF138302	DCN	decorin
OC	201893_x_at	1634	AF138300	DCN	decorin
OC	211813_x_at	1634	AF138303	DCN	decorin
OC	203699_s_at	1734	U53506	DIO2	deiodinase, iodothyronine, type II
OC	225809_at	25849	AI659927	DKFZP564O0823	DKFZP564O0823 protein
OC	232090_at	26052	AI761578	DNM3	Dynamin 3
OC	213071_at	1805	AL049798	DPT	dermatopontin
OC	213068_at	1805	AI146848	DPT	dermatopontin
OC	206101_at	1842	NM_001393	ECM2	extracellular matrix protein 2, female organ and adipocyte specific
OC	219436_s_at	51705	NM_016242	EMCN	endomucin
OC	204160_s_at	22875	AW194947	ENPP4	ectonucleotide pyrophosphatase/phosphodiesterase 4 (putative function)
OC	228585_at	404033	AI301948	ENTPD1	Ectonucleoside triphosphate diphosphohydrolase 1
OC	228256_s_at	114915	AU144565	EPB41L4A	erythrocyte membrane protein band 4.1 like 4A
OC	1554547_at	220965	BC036453	FAM13C1	family with sequence similarity 13, member C1
OC	241981_at	54757	AW291369	FAM20A	family with sequence similarity 20, member A
OC	226804_at	54757	AI632223	FAM20A	family with sequence similarity 20, member A
OC	242945_at	54757	AI860568	FAM20A	family with sequence similarity 20, member A
OC	202995_s_at	2192	NM_006486	FBLN1	fibulin 1
OC	202994_s_at	2192	Z95331	FBLN1	fibulin 1
OC	235318_at	---	AW955612	FBN1	fibrillin 1
OC	222245_s_at	80307	AF218012	FER1L4	fer-1-like 4 (C. elegans)
OC	223620_at	2857	AF039686	GPR34	G protein-coupled receptor 34
OC	202957_at	3059	NM_005335	HCLS1	hematopoietic cell-specific Lyn substrate 1
OC	235944_at	83872	BF446673	HMCN1	hemicentin 1
OC	209292_at	3400	AL022726	ID4	Inhibitor of DNA binding 4, dominant negative helix-loop-helix protein
OC	209291_at	3400	AW157094	ID4	inhibitor of DNA binding 4, dominant negative helix-loop-helix protein
OC	228518_at	3500	AW575313	IGHM	immunoglobulin heavy constant mu
OC	202747_s_at	9452	NM_004867	ITM2A	integral membrane protein 2A
OC	202746_at	9452	AL021786	ITM2A	integral membrane protein 2A
OC	219213_at	58494	NM_021219	JAM2	junctional adhesion molecule 2
OC	206478_at	9834	NM_014792	KIAA0125	KIAA0125
OC	206481_s_at	9079	NM_001290	LDB2	LIM domain binding 2
OC	228058_at	124220	AI559190	LOC124220	similar to common salivary protein 1
OC	229130_at	285535	AU145323	LOC285535	hypothetical protein LOC285535
OC	203548_s_at	4023	BF672975	LPL	lipoprotein lipase
OC	213909_at	131578	AU147799	LRRC15	leucine rich repeat containing 15
OC	202018_s_at	4057	NM_002343	LTF	lactotransferrin
OC	202350_s_at	4147	NM_002380	MATN2	matrilin 2
OC	206201_s_at	4223	NM_005924	MEOX2	mesenchyme homeobox 2
OC	207761_s_at	25840	NM_014033	METTL7A	methyltransferase like 7A
OC	212713_at	4239	R72286	MFAP4	microfibrillar-associated protein 4
OC	229254_at	148808	BE550027	MFSD4	major facilitator superfamily domain containing 4
OC	232568_at	54796	AU145658	MGC24103	hypothetical protein MGC24103
OC	202291_s_at	4256	NM_000900	MGP	matrix Gla protein

OC	213693_s_at	4582	AI610869	MUC1	mucin 1, cell surface associated
OC	217110_s_at	4585	AJ242547	MUC4	mucin 4, cell surface associated
OC	217109_at	4585	AJ242547	MUC4	mucin 4, cell surface associated
OC	204895_x_at	4585	NM_004532	MUC4	mucin 4, cell surface associated
OC	227870_at	57722	AB046848	NOPE	likely ortholog of mouse neighbor of Punc E11
OC	221210_s_at	80896	NM_030769	NPL	N-acetylneuraminate pyruvate lyase (dihydrodipicolinate synthase)
OC	205591_at	10439	NM_006334	OLFM1	olfactomedin 1
OC	218162_at	56944	NM_020190	OLFML3	olfactomedin-like 3
OC	205908_s_at	4958	NM_005014	OMD	osteomodulin
OC	226435_at	89932	AU145309	PAPLN	papilin, proteoglycan-like sulfated glycoprotein
OC	212151_at	5087	BF967998	PBX1	Pre-B-cell leukemia transcription factor 1
OC	217617_at	5087	AW451711	PBX1	Pre-B-cell leukemia transcription factor 1
OC	212148_at	5087	AL049381	PBX1	Pre-B-cell leukemia transcription factor 1
OC	205226_at	5157	NM_006207	PDGFRL	platelet-derived growth factor receptor-like
OC	208982_at	5175	AW574504	PECAM1	Platelet/endothelial cell adhesion molecule (CD31 antigen)
OC	208983_s_at	5175	M37780	PECAM1	platelet/endothelial cell adhesion molecule (CD31 antigen)
OC	222288_at	389129	AI004009	PPP4R2	Protein phosphatase 4, regulatory subunit 2
OC	207177_at	5737	NM_000959	PTGFR	prostaglandin F receptor (FP)
OC	209496_at	5919	BC000069	RARRES2	retinoic acid receptor responder (tazarotene induced) 2
OC	218353_at	8490	NM_025226	RGS5	regulator of G-protein signalling 5
OC	209070_s_at	8490	AI183997	RGS5	regulator of G-protein signalling 5
OC	209071_s_at	8490	AF159570	RGS5	regulator of G-protein signalling 5
OC	1555725_a_at	8490	AF493929	RGS5	regulator of G-protein signalling 5
OC	235489_at	57381	AI583530	RHOJ	ras homolog gene family, member J
OC	201785_at	6035	NM_002933	RNASE1	ribonuclease, RNase A family, 1 (pancreatic)
OC	205529_s_at	862	NM_004349	RUNX1T1	runt-related transcription factor 1; translocated to, 1 (cyclin D-related)
OC	214456_x_at	6289	M23699	SAA1	serum amyloid A1
OC	208607_s_at	6289	NM_030754	SAA1	serum amyloid A1
OC	235849_at	286133	BE787752	SCARA5	scavenger receptor class A, member 5 (putative)
OC	229839_at	286133	AI799784	SCARA5	Scavenger receptor class A, member 5 (putative)
OC	206211_at	6401	NM_000450	SELE	selectin E (endothelial adhesion molecule 1)
OC	214433_s_at	8991	NM_003944	SELENBP1	selenium binding protein 1 /// selenium binding protein 1
OC	206049_at	6403	NM_003005	SELP	selectin P (granule membrane protein 140kDa, antigen CD62)
OC	201427_s_at	6414	NM_005410	SEPP1	selenoprotein P, plasma, 1
OC	202283_at	5176	NM_002615	SERPINF1	serpin peptidase inhibitor, clade F (alpha-2 antiplasmin, pigment epithelium derived factor), member 1
OC	223122_s_at	6423	AF311912	SFRP2	secreted frizzled-related protein 2
OC	221024_s_at	81031	NM_030777	SLC2A10	solute carrier family 2 (facilitated glucose transporter), member 10
OC	223235_s_at	64094	AB014737	SMOC2	SPARC related modular calcium binding 2
OC	219511_s_at	9627	NM_005460	SNCAIP	synuclein, alpha interacting protein (synphilin)
OC	225728_at	8470	AI659533	SORBS2	sorbin and SH3 domain containing 2
OC	200795_at	8404	NM_004684	SPARCL1	SPARC-like 1 (mast9, hevin)
OC	201998_at	6480	AI743792	ST6GAL1	ST6 beta-galactosamide alpha-2,6-sialyltranferase 1
OC	38487_at	23166	D87433	STAB1	stabilin 1
OC	204150_at	23166	NM_015136	STAB1	stabilin 1
OC	225895_at	171024	AI634580	SYNPO2	synaptopodin 2
OC	227662_at	171024	AA541622	SYNPO2	synaptopodin 2
OC	204731_at	7049	NM_003243	TGFBR3	transforming growth factor, beta receptor III (betaglycan, 300kDa)
OC	210166_at	7100	AF051151	TLR5	toll-like receptor 5
OC	227300_at	338773	AL521682	TMEM119	transmembrane protein 119
OC	235735_at	944	AI936516	TNFSF8	Tumor necrosis factor (ligand) superfamily, member 8
OC	240174_at	388903	BF512871	TNRC6B	Trinucleotide repeat containing 6B
OC	219274_at	23554	NM_012338	TSPAN12	tetraspanin 12
OC	201009_s_at	10628	AI439556	TXNIP	thioredoxin interacting protein
OC	201008_s_at	10628	AA812232	TXNIP	thioredoxin interacting protein
OC	201010_s_at	10628	NM_006472	TXNIP	thioredoxin interacting protein
OC	204787_at	11326	NM_007268	VSIG4	V-set and immunoglobulin domain containing 4
OC	202112_at	7450	NM_000552	VWF	von Willebrand factor
OC	226676_at	25925	AK021452	ZNF521	zinc finger protein 521
OC	226677_at	25925	AF141339	ZNF521	zinc finger protein 521

Table S2: Details regarding the 41 genes with a strong fold change in their expression (an absolute fold change of 1.8 i.e. 0.85 Log2 scale, and a corrected *P* for multiple testing with a Benjamini-Hochberg test (BH) below 5%).

Symbol	Entrez Gene ID	High in	p-value	p-value BH
**Inflammation**				
ADAMDEC1	27299	OC	***	*
APOC1	341	OC	***	*
C3	718	OC	***	*
CD163	1631	OC	***	**
ACKR1	4035	OC	***	**
VSIG4	11326	OC	***	**
**Oncologic processes signaling**				
TXNIP	10628	OC	***	*
TGFA	7039	BC	***	*
SCARA5	286133	OC	***	*
LPAR3	23566	BC	***	*
FGFBP1	9982	BC	**	*
CTHRC1	115908	OC	***	*
SFRP2	6423	OC	***	*
MGP	4256	OC	***	**
RGS5	8490	OC	***	*
VWF	7450	OC	***	*
**Extracellular matrix**				
COL3A1	1281	OC	***	*
CLEC3B	7123	OC	***	**
SELP	6403	OC	***	**
DCN	1634	OC	***	*
SPARCL1	8404	OC	***	*
**EMT&CAF**				
CXCL12	6387	OC	***	*
ZNF521	25925	OC	***	*
OLFML3	56944	OC	***	*
OLFM1	10439	OC	***	**
ASPN	54829	OC	***	*
**Cell metabolisms**				
ELAVL2	1993	Down	***	*
IRF6	3664	Down	***	*
OCIAD2	132299	Down	***	*
PLEK2	26499	Down	***	*
CYP1B1	1545	Up	***	*
ANXA1	301	Down	***	*
FST	10468	Down	***	*
RBP1	5947	Down	***	**
CARD18	59082	Down	***	*
RHOD	29984	Down	***	*
PAQR5	54852	Down	***	*
VSNL1	7447	Down	***	*
ODC1	4953	Down	***	*
UPP1	7378	Down	***	*
RGS20	8601	Down	***	**

Table S3: Details regarding the genes represented in Figure 2F

Symbol	Type	Entrez Gene ID	Name
ACSS1	enriched	84532	acyl-CoA synthetase short-chain family member 1
ACTN1	enriched	87	actinin, alpha 1
AKAP2	enriched	11217	A kinase (PRKA) anchor protein 2
ANXA6	enriched	309	annexin A6
BICC1	enriched	80114	BicC family RNA binding protein 1
BPTF	enriched	2186	bromodomain PHD finger transcription factor
C11orf96	enriched	387763	chromosome 11 open reading frame 96
CAV1	enriched	857	caveolin 1, caveolae protein, 22kDa
CDCA7L	enriched	55536	cell division cycle associated 7-like
CRISPLD2	enriched	83716	cysteine-rich secretory protein LCCL domain containing 2
CSGALNACT2	enriched	55454	chondroitin sulfate N-acetylgalactosaminyltransferase 2
DAB2	enriched	1601	Dab, mitogen-responsive phosphoprotein, homolog 2 (Drosophila)
DCN	enriched	1634	decorin
ETS1	enriched	2113	v-ets avian erythroblastosis virus E26 oncogene homolog 1
FAM117A	enriched	81558	family with sequence similarity 117, member A
FAT4	enriched	79633	FAT atypical cadherin 4
FLJ44635	enriched	392490	TPT1-like protein
FNDC1	enriched	84624	fibronectin type III domain containing 1
FSTL1	enriched	11167	follistatin-like 1
FYN	enriched	2534	FYN proto-oncogene, Src family tyrosine kinase
GALNT15	enriched	117248	polypeptide N-acetylgalactosaminyltransferase 15
GAS1	enriched	2619	growth arrest-specific 1
GPM6B	enriched	2824	glycoprotein M6B
GPR124	enriched	25960	G protein-coupled receptor 124
GUCY1B3	enriched	2983	guanylate cyclase 1, soluble, beta 3
HECW2	enriched	57520	HECT, C2 and WW domain containing E3 ubiquitin protein ligase 2
IQSEC1	enriched	9922	IQ motif and Sec7 domain 1
KDELC1	enriched	79070	KDEL (Lys-Asp-Glu-Leu) containing 1
KIRREL	enriched	55243	kin of IRRE like (Drosophila)
KLF7	enriched	8609	Kruppel-like factor 7 (ubiquitous)
LAMA4	enriched	3910	laminin, alpha 4
LEF1	marker	51176	lymphoid enhancer-binding factor 1
LIX1L	enriched	128077	Lix1 homolog (chicken) like
MASP1	enriched	5648	mannan-binding lectin serine peptidase 1 (C4/C2 activating component of Ra-reactive factor)
MSI2	enriched	124540	musashi RNA-binding protein 2
PALM2-AKAP2	enriched	445815	PALM2-AKAP2 readthrough
PDGFRA	enriched	5156	platelet-derived growth factor receptor, alpha polypeptide
PDGFRB	enriched	5159	platelet-derived growth factor receptor, beta polypeptide
PDPN	enriched	10630	podoplanin
PID1	enriched	55022	phosphotyrosine interaction domain containing 1
PIK3R3	enriched	8503	phosphoinositide-3-kinase, regulatory subunit 3 (gamma)
PLXNC1	enriched	10154	plexin C1
PMP22	enriched	5376	peripheral myelin protein 22
PRRX1	enriched	5396	paired related homeobox 1
PTPRM	enriched	5797	protein tyrosine phosphatase, receptor type, M
PXYLP1	enriched	92370	2-phosphoxylose phosphatase 1
SAMD4A	enriched	23034	sterile alpha motif domain containing 4A
SEMA3C	enriched	10512	sema domain, immunoglobulin domain (Ig), short basic domain, secreted, (semaphorin) 3C
SMOC2	enriched	64094	SPARC related modular calcium binding 2
SMTN	enriched	6525	smoothelin
SNAI1	marker	6615	snail family zinc finger 1
SNAI2	marker	6591	snail family zinc finger 2
SYNE1	enriched	23345	spectrin repeat containing, nuclear envelope 1
TCF4	enriched	6925	transcription factor 4
TIMP1	enriched	7076	TIMP metallopeptidase inhibitor 1
TNC	enriched	3371	tenascin C
TNFRSF12A	enriched	51330	tumor necrosis factor receptor superfamily, member 12A
TUBA1A	enriched	7846	tubulin, alpha 1a
TWIST1	marker	7291	twist family bHLH transcription factor 1
TWIST2	marker	117581	twist family bHLH transcription factor 2
VIM	marker	7431	vimentin
ZCCHC24	enriched	219654	zinc finger, CCHC domain containing 24
ZEB1	marker	6935	zinc finger E-box binding homeobox 1
ZEB2	enriched	9839	zinc finger E-box binding homeobox 2
ZNF362	enriched	149076	zinc finger protein 362

Table S4: Details regarding the genes represented in Figure 4F

Symbol	Type	Entrez Gene ID	Name
ACTA2	marker	59	actin, alpha 2, smooth muscle, aorta
ADAMTS12	enriched	81792	ADAM metallopeptidase with thrombospondin type 1 motif, 12
ADAMTS2	enriched	9509	ADAM metallopeptidase with thrombospondin type 1 motif, 2
ARHGAP26	enriched	23092	Rho GTPase activating protein 26
C7orf63	enriched	79846	-
CDC27	enriched	996	cell division cycle 27
COL1A2	enriched	1278	collagen, type I, alpha 2
COL5A1	enriched	1289	collagen, type V, alpha 1
COL5A2	enriched	1290	collagen, type V, alpha 2
COL5A3	enriched	50509	collagen, type V, alpha 3
COL6A1	enriched	1291	collagen, type VI, alpha 1
CXCL12	marker	6387	chemokine (C-X-C motif) ligand 12
EBF1	enriched	1879	early B-cell factor 1
EFEMP2	enriched	30008	EGF containing fibulin-like extracellular matrix protein 2
EHD2	enriched	30846	EH-domain containing 2
EMILIN2	enriched	84034	elastin microfibril interfacer 2
FAP	marker	2191	fibroblast activation protein, alpha
FSTL1	enriched	11167	follistatin-like 1
FYN	enriched	2534	FYN proto-oncogene, Src family tyrosine kinase
FZD4	enriched	8322	frizzled class receptor 4
GLIS3	enriched	169792	GLIS family zinc finger 3
GUCY1A3	enriched	2982	guanylate cyclase 1, soluble, alpha 3
GUCY1B3	enriched	2983	guanylate cyclase 1, soluble, beta 3
IDNK	enriched	414328	idnK, gluconokinase homolog (E. coli)
KIF23	enriched	9493	kinesin family member 23
LAMA4	enriched	3910	laminin, alpha 4
LAMB1	enriched	3912	laminin, beta 1
LOC389906	enriched	389906	-
MIR143HG	enriched	728264	MIR143 host gene (non-protein coding)
MYL9	enriched	10398	myosin, light chain 9, regulatory
NICN1	enriched	84276	nicolin 1
NID2	enriched	22795	nidogen 2 (osteonidogen)
OLFML2B	enriched	25903	olfactomedin-like 2B
PCDH18	enriched	54510	protocadherin 18
PDE3A	enriched	5139	phosphodiesterase 3A, cGMP-inhibited
PDE4A	enriched	5141	phosphodiesterase 4A, cAMP-specific
PDGFRB	marker	5159	platelet-derived growth factor receptor, beta polypeptide
PDLIM4	enriched	8572	PDZ and LIM domain 4
PDPN	enriched	10630	podoplanin
PRRX1	enriched	5396	paired related homeobox 1
PTRF	enriched	284119	polymerase I and transcript release factor
RBM5	enriched	10181	RNA binding motif protein 5
RUNX1T1	enriched	862	runt-related transcription factor 1; translocated to, 1 (cyclin D-related)
S100A4	marker	6275	S100 calcium binding protein A4
SDC2	enriched	6383	syndecan 2
SLC16A10	enriched	117247	solute carrier family 16 (aromatic amino acid transporter), member 10
SPARC	enriched	6678	secreted protein, acidic, cysteine-rich (osteonectin)
TAGLN	enriched	6876	transgelin
TGFB1	marker	7040	transforming growth factor, beta 1
TMEM204	enriched	79652	transmembrane protein 204
TSHZ3	enriched	57616	teashirt zinc finger homeobox 3
UFD1L	enriched	7353	ubiquitin fusion degradation 1 like (yeast)
VGLL3	enriched	389136	vestigial-like family member 3
WDR4	enriched	10785	WD repeat domain 4
WDR48	enriched	57599	WD repeat domain 48
ZBTB46	enriched	140685	zinc finger and BTB domain containing 46
ZEB1	enriched	6935	zinc finger E-box binding homeobox 1
ZEB2	enriched	9839	zinc finger E-box binding homeobox 2
